# Ivermectin reduces in vivo coronavirus infection in a mouse experimental model

**DOI:** 10.1038/s41598-021-86679-0

**Published:** 2021-03-30

**Authors:** A. P. Arévalo, R. Pagotto, J. L. Pórfido, H. Daghero, M. Segovia, K. Yamasaki, B. Varela, M. Hill, J. M. Verdes, M. Duhalde Vega, M. Bollati-Fogolín, M. Crispo

**Affiliations:** 1 Transgenic and Experimental Animal Unit, Institut Pasteur de Montevideo, Montevideo, Uruguay; 2 Cell Biology Unit, Institut Pasteur de Montevideo, Montevideo, Uruguay; 3 Worm Biology Laboratory, Institut Pasteur de Montevideo/Department of Biosciences, Faculty of Chemistry, University of the Republic, Montevideo, Uruguay; 4 Laboratory of Immunoregulation and Inflammation, Institut Pasteur de Montevideo, Montevideo, Uruguay; 5 Immunobiology Department, Faculty of Medicine, University of the Republic, Montevideo, Uruguay; 6 Pathobiology Department, Faculty of Veterinary, Pathology Unit, University of the Republic, Montevideo, Uruguay; 7 Institute of Biological Chemistry and Chemical Physics (UBA-CONICET), School of Pharmacy and Biochemistry, University of Buenos Aires, Buenos Aires, Argentina

**Keywords:** Diseases, Signs and symptoms

## Abstract

The objective of this study was to test the effectiveness of ivermectin for the treatment of mouse hepatitis virus (MHV), a type 2 family RNA coronavirus similar to SARS-CoV-2. Female BALB/cJ mice were infected with 6,000 PFU of MHV-A59 (group infected, n = 20) or infected and then immediately treated with a single dose of 500 µg/kg ivermectin (group infected + IVM, n = 20) or were not infected and treated with PBS (control group, n = 16). Five days after infection/treatment, the mice were euthanized and the tissues were sampled to assess their general health status and infection levels. Overall, the results demonstrated that viral infection induced typical MHV-caused disease, with the livers showing severe hepatocellular necrosis surrounded by a severe lymphoplasmacytic inflammatory infiltration associated with a high hepatic viral load (52,158 AU), while mice treated with ivermectin showed a better health status with a lower viral load (23,192 AU; p < 0.05), with only a few having histopathological liver damage (p < 0.05). No significant differences were found between the group infected + IVM and control group mice (P = NS). Furthermore, serum transaminase levels (aspartate aminotransferase and alanine aminotransferase) were significantly lower in the treated mice than in the infected animals. In conclusion, ivermectin diminished the MHV viral load and disease in the mice, being a useful model for further understanding this therapy against coronavirus diseases.

## Introduction

Mouse hepatitis virus (MHV) is a single-stranded RNA coronavirus that targets different organs^1^. The virus is highly contagious, has natural respiratory or oral transmission, and shows high morbidity and low mortality rates. There is no vaccine or treatment available; therefore, upon infection, an entire laboratory mouse colony must be sacrificed to control the disease.

Recent studies have shown that the mechanism of infection has similarities to that of SARS-CoV-2^2,3^: therefore, it has been proposed that MHV may be an interesting infection model to test new therapies against COVID-19 in animals. Although different therapies have been evaluated, no effective treatment is available, and the mechanism by which the virus enters the cell is being explored^4^. After entry into the cytoplasm of the host cell, coronaviruses rely on a nuclear transport system mediated by the importin α/β1 heterodimer to facilitate replication and infection^5,6^. Some drugs have been demonstrated to act by impairing importin α/β1 heterodimer formation to prevent viral entry. Because both MHV and SARS-CoV-2 enter the nucleus via the same mechanism, MHV may be an interesting target for the development of candidate therapies against coronavirus infection in a mouse model.

Ivermectin is an efficient and inexpensive drug usually applied to treat parasitic infestations. It has been approved by the FDA for animal and human use and is available worldwide. It has a wide margin of safety with an LD_50_ of 30 mg/kg in mice and is used in humans as an antiparasitic treatment at a dose of 150–200 µg/kg^7^. Other effects of this drug, such as antimicrobial^8^, anti-trypanosome/anti-malaria^9^ and anticancer activities^10^, have been proposed.

In addition, several reports have shown an in vitro effect of ivermectin against RNA and DNA virus infection through the suppression of the host cellular process related to the inhibition of the nuclear transport of specific proteins required for viral replication^11^. Exposure to ivermectin has shown some positive effects against almost 20 different types of viruses, including Zika, dengue and chikungunya, indicating a broad range of action and potential applications^12^.

Although an effect of ivermectin against coronaviruses has been proposed in previous reports, decreased viral load and improved clinical parameters have yet to be demonstrated in vivo. Recently, it was reported in an in vitro cell model that ivermectin was effective against SARS-CoV-2, showing an inhibition of virus replication, making it a repurposing candidate to treat COVID-19^13^. Scientific information on the in vivo antiviral effect of ivermectin against coronavirus is still scarce, with only a few preclinical models such as the golden Syrian hamster^14^ reported thus far. Moreover, several observational and randomized clinical trials are currently being conducted to gain deeper knowledge about the effect of this drug alone or in combination with other drugs on SARS-CoV-2^15–22^, but more data are needed before the development of new therapeutic strategies for the control of these types of coronaviruses can be promoted.

The objective of this study was to evaluate the general health profile, hepatic viral load and functionality of ivermectin in vivo for the treatment of a type 2 family RNA coronavirus, MHV, in a murine model. We hypothesize that the administration of a single dose of ivermectin to recently infected mice will decrease the viral load and impair the action of the virus against the host organism.

## Results

### Body weight and macroscopic liver appearance were impaired in infected mice not treated with ivermectin

**Figure 1:**
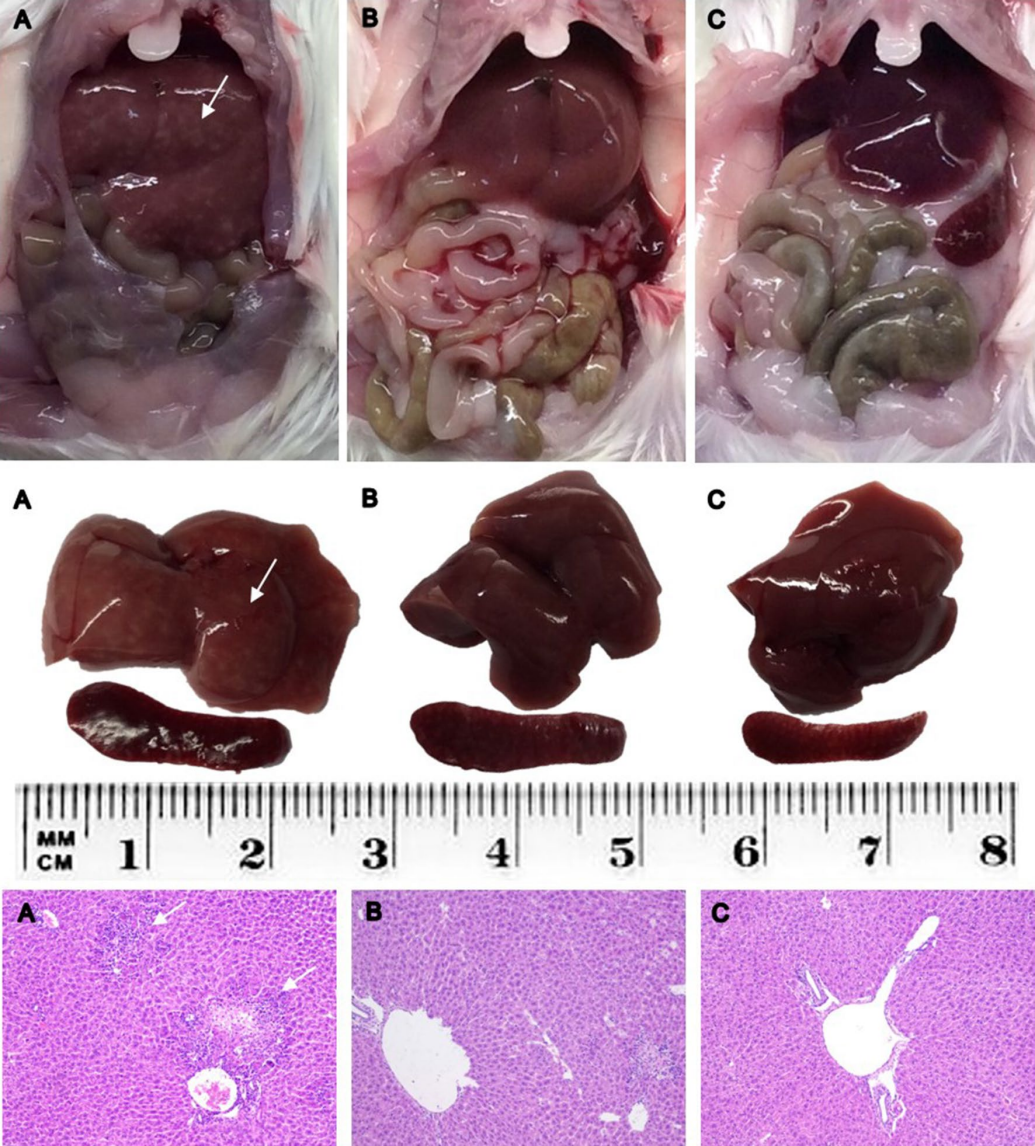
Representative liver and spleen from each group: (**A**) infected; (**B**) infected + IVM; (**C**) control. Upper panel: abdominal cavity at necropsy; middle panel: dissected liver and spleen; lower panel: HE histological sections of livers. White arrows indicate white spotted patterns in the liver from infected mice and severe hepatocellular necrosis and lymphoplasmacytic inflammatory infiltration in the histological images (**A**). *IVM* ivermectin.

**Figure 2:**
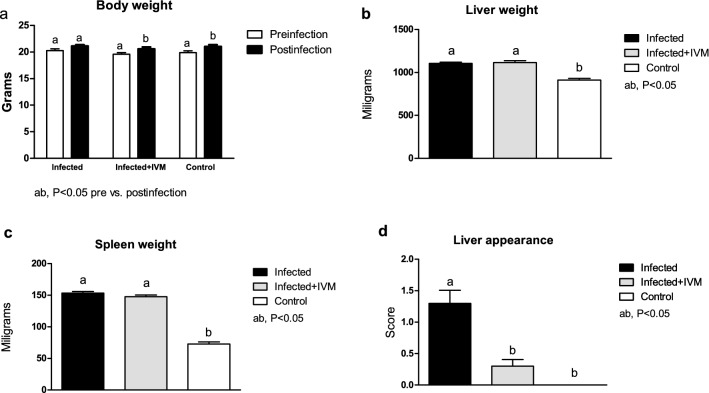
Body weight at the beginning and the end of the experiment (**a**) and organ weight and liver appearance at necropsy five days postinfection (**b**,**c**,**d**, respectively) in MHV-infected (infected group, n = 20), infected and treated with ivermectin (infected + IVM group, n = 20), and noninfected untreated mice (control group, n = 16) (mean ± SD). Different letters indicate significant differences (p < 0.05) between pre- and postinfection timepoints for a and significant differences (p < 0.05) between the indicated groups for (**b**–**d**).

Viral infection had an effect on mouse body weight pre- and postinfection, with animals from the control and infected + IVM groups gaining weight (p < 0.05), and mice from the infected group showing no significant differences pre- and postinfection (P = NS). Both the livers and spleens from the infected and infected + IVM group mice were heavier than those from the control group at necropsy (p < 0.05). The macroscopic liver appearance was impaired in the infected group compared to the infected + IVM and control groups (p < 0.05). These results are shown in Figs. 1 and 2.

### Ivermectin enhances histopathological liver scores in infected mice

**Figure 3:**
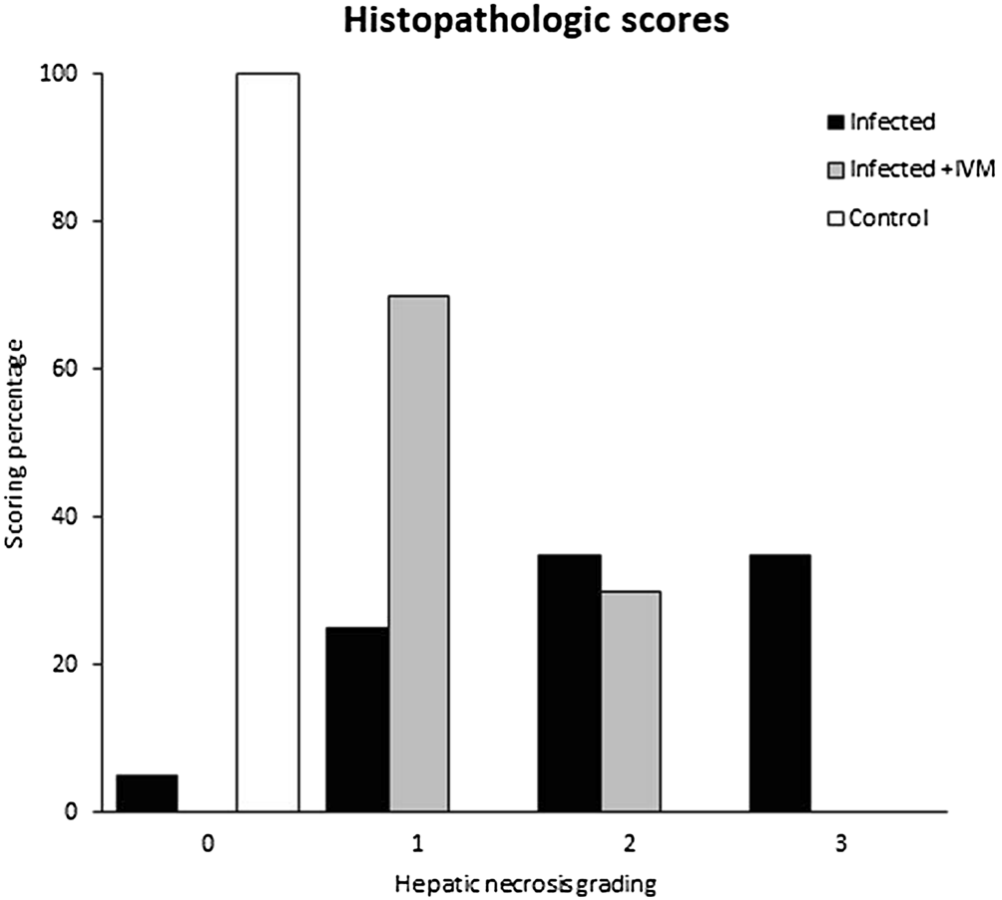
Histopathological scores (grades 0 to 3, where 0 is no damage and 3 is the most damaged) of livers from the MHV-infected (infected group, n = 20), infected and treated with ivermectin (infected + IVM group, n = 20), and noninfected untreated mice (control group, n = 16). The distribution of animals for each score showed significant differences between the groups (p < 0.05).

Histopathological scores (mean ± S.D. and median) were 2.00 ± 0.92 and 2 for the infected group (n = 20), 1.30 ± 0.47 and 1 for the infected + IVM group (n = 20), and 0 for the control group (n = 16). The scoring percentage for each necrotic grade is shown in Fig. 3. Briefly, the livers of the mice from the infected group were characterized by severe hepatocellular necrosis, with a high number of specimens with more than 10 necrotic areas surrounded by severe lymphoplasmacytic inflammatory infiltration. Typical hepatocellular necrosis and inflammatory infiltration were present but with less severity in the livers of the mice in the infected + IVM group (p < 0.05). The livers of the mice from the control group did not show any hepatocellular or spleen lesions. Representative histological liver images are shown in Fig. 1.

### A single dose of ivermectin reduces the viral load in infected mice

**Figure 4:**
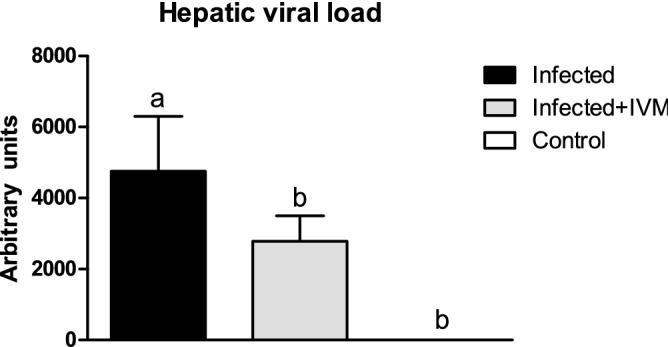
Hepatic viral load measured by qPCR in liver samples from MHV-infected (infected group, n = 20), infected and treated with ivermectin (infected + IVM group, n = 20), and noninfected untreated mice (control group, n = 16) (mean ± SD). Different letters indicate significant differences (p < 0.05) among the groups.

The results obtained from qPCR analysis showed a significantly higher viral load in the livers of the infected group compared to that in the livers of the infected + IVM and control groups (p < 0.05), with a load of 52,158 ± 15,235 arbitrary units (AU) for the infected animals (Fig. 4). Ivermectin treatment significantly lowered the viral load (23,192 ± 6796 AU; p < 0.05), which is in accordance with its effects on other disease features that were observed.

### Viral infection affects liver and kidney protein profiles

**Figure 5:**
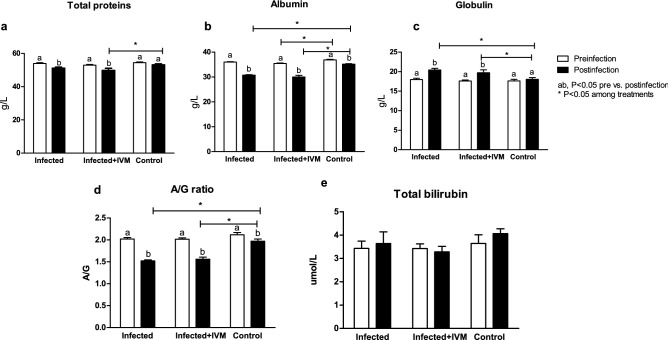
Protein levels measured in the blood of mice in the MHV-infected (infected group, n = 20), infected and treated with ivermectin (infected + IVM group, n = 20), and noninfected untreated mice (control group, n = 16) before and after infection with MHV A-59. (**a**) Total proteins; (**b**) albumin; (**c**) globulin, (**d**) albumin/globulin ratio and (**e**) total bilirubin (mean ± SD). Different letters indicate significant differences (p < 0.05) between the pre- and post-infection timepoints; asterisks (*) refer to significant differences (p < 0.05) between indicated groups.

To assess the liver and kidney health profiles, several biochemical parameters were analyzed pre- and postinfection from complete blood, such as total protein (TP) level; albumin (ALB) level, globulin (GLO) level, ALB/GLO ratio, total bilirubin (TBIL) level, alanine aminotransferase (ALT) level, aspartate aminotransferase (AST) level, gamma glutamyl transpeptidase (GGT) level, blood urea nitrogen (BUN) level, creatinine (CRE), BUN/CRE ratio and glucose (GLU) level. The total protein level decreased after infection in the mice from the infected and infected + IVM groups compared with their basal profile level (p < 0.05) (Fig. 5a). The albumin levels decreased in the mice in all of the experimental groups and were lower in the infected and infected + IVM groups (p < 0.05) (Fig. 5b). In contrast, the globulin levels increased after infection in both infected groups, showing significant differences compared to the control group (p < 0.05) (Fig. 5c). The A/G ratio decreased between the basal and final stages in the three groups, and it was lower for both infected groups (p < 0.05) (Fig. 5d). The total bilirubin level was significantly different between the basal and final measurements among the groups (Fig. 5e).

### Ivermectin decreases the hepatic transaminase levels in infected mice

**Figure 6:**
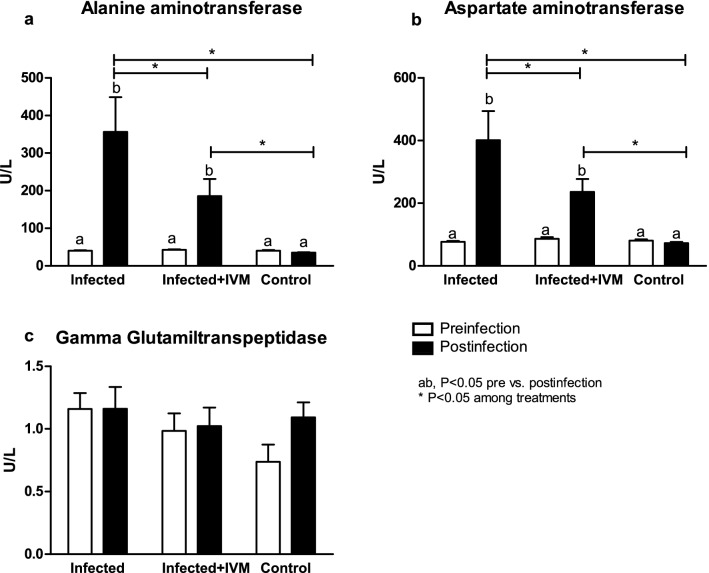
Hepatic enzyme levels in the blood of MHV-infected (infected group, n = 20), infected and treated with ivermectin (infected + IVM group, n = 20), and noninfected untreated mice (control group, n = 16) before and after virus infection. (**a**) Alanine aminotransferase; (**b**) aspartate aminotransferase and (**c**) gamma glutamyl transpeptidase (mean ± SD). Different letters indicate significant differences (p < 0.05) between pre- and postinfection timepoints; asterisks (*) refer to significant differences (p < 0.05) between the indicated groups.

**Figure 7:**
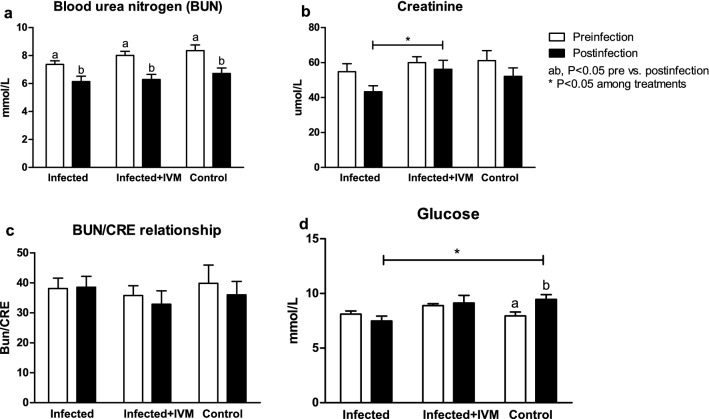
Blood urea nitrogen (BUN), creatinine (CRE), BUN/CRE ratio and glucose (GLU) levels were measured before and after infection in MHV-infected (infected group, n = 20), infected and treated with ivermectin (infected + IVM group, n = 20), and noninfected untreated mice (control group, n = 16). The results are shown in (**a**–**d**) (mean ± SD). Different letters indicate significant differences (p < 0.05) between pre- and postinfection timepoints; asterisks (*) refer to significant differences (p < 0.05) between the indicated groups.

Hepatic transaminases such as ALT and AST showed an important increase in the animals from the infected group compared with the animals from the infected + IVM and control groups (p < 0.05) (Fig. 6a,b). The GGT levels were similar between the groups pre- and postinfection (P = NS) (Fig. 6c). The BUN levels were decreased after infection/treatment (p < 0.05). The CRE levels were lower in the animals in the infected group at the end of the experiment than in the animals in the infected + IVM group (p < 0.05). The BUN/CRE ratio was similar between the groups at all timepoints (P = NS), but the GLU levels were decreased in the animals from the infected group after infection compared with those in the control group (p < 0.05). The results are shown in Fig. 7a–d.

### Neutrophil and monocyte blood levels were increased in the infected animals

**Figure 8:**
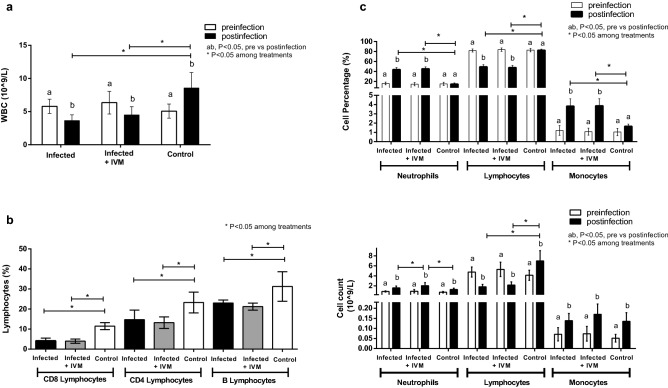
Evaluation of hematological parameters in mice. (**a**) White blood cell (WBC) count, (**c**,**d**) neutrophil, lymphocyte and monocyte counts and percentages were determined in blood samples before and after infection for each experimental group (infected, infected + IVM, and control) (mean ± SD). (**b**) Lymphocyte staining for the expression of surface markers for B and T cells at the end point of the experiment for all groups (infected, infected + IVM, and control) (mean ± SD). Different letters indicate significant differences (p < 0.05) between pre- and postinfection timepoints; asterisks (*) refer to significant differences (p < 0.05) between the indicated groups.

The hematological parameters measured in the peripheral blood samples pre- and postinfection are summarized in Table 1. A significant decrease in the number of white blood cells (WBCs) was found in the animals from both virus-infected groups (Fig. 8a), whereas no differences in number of red blood cells (RBCs), hemoglobin (HGC) level, hematocrit (HCT) level, or number of platelets were found compared with these measures in the control and preinfection groups (Table 1). Absolute WBC and lymphocyte counts were increased in the control group compared to those at the pre- and postinfection timepoints.

**Table 1:** Hematological parameters from peripheral blood samples of the three experimental groups, measured pre- and postinfection.

	Infected		Infected + IVM		Control	
Parameter	Pre	Pos	Pre	Pos	Pre	Pos
WBC # (10^9^/L)	5.80 (1.07)	3.64 (0.87)	6.35 (1.71)	4.49 (1.25)	5.07 (1.07)	8.56 (2.32)
Neu # (10^9^/L)	0.89 (0.12)	1.59 (0.38)	0.92 (0.33)	2.03 (0.62)	0.742 (0.15)	1.28 (0.25)
Lym # (10^9^/L)	4.77 (1.00)	1.80 (0.49)	5.31 (1.44)	2.15 (0.64)	4.12 (1.02)	6.99 (2.05)
Mon # (10^9^/L)	0.07 (0.03)	0.14 (0.03)	0.07 (0.04)	0.17 (0.05)	0.05 (0.02)	0.13 (0.04)
Eos # (10^9^/L)	0.05 (0.02)	0.05 (0.04)	0.04 (0.03)	0.06 (0.02)	0.05 (0.02)	0.08 (0.03)
Bas # (10^9^/L)	0.01 (0.01)	0.06 (0.02)	0.01 (0.01)	0.08 (0.02)	0.01 (0.01)	0.02 (0.01)
Neu % (%)	15.9 (2.8)	43.6 (4.0)	14.4 (3.3)	45.1 (4.0)	15.2 (3.3)	21.4 (12.7)
Lymp % (%)	81.8 (3.0)	49.2 (4.2)	83.6 (3.5)	47.7 (4.3)	82.2 (3.5)	74.9 (14.2)
Mon % (%)	1.2 (0.5)	3.8 (0.8)	1.1 (0.4)	3.8 (0.7)	1.1 (0.3)	1.5 (0.2)
Eos % (%)	0.8 (0.5)	1.4 (0.4)	0.6 (0.3)	1.3 (0.7)	1.1 (0.4)	1.0 (0.4)
Bas % (%)	0.2 (0.1)	1.8 (0.4)	0.2 (0.0)	1.9 (0.4)	0.3 (0.2)	0.2 (0.1)
RBC (10^12^/L)	10.0 (0.7)	8.4 (0.6)	10.3 (1.3)	9.0 (0.8)	10.2 (1.1)	8.5 (0.8)
HGC (g/L)	167 (14)	143 (10)	171 (22)	153 (14)	171 (18)	148 (16)
HCT (%)	48.9 (3.7)	42.0 (3.0)	50.3 (6.4)	45.4 (4.2)	49.6 (5.2)	41.7 (4.1)
PLT (10^9^/L)	620 (184)	831 (198)	675 (209)	765 (251)	647 (204)	757 (196)

Data are expressed as the means (SD).

*Neu* neutrophils; *Lym* lymphocytes; *Mon* monocytes; *Eos* eosinophils; *Bas* basophils; *RBC* red blood cells; *HGB* hemoglobin; *HCT* hematocrit; *PLT* platelets.

Measurements of the WBC differences revealed that the percentage of neutrophils and monocytes were significantly increased in animals in both infected groups. In contrast, the percentage and counts of lymphocytes were the only WBC parameters that were significantly decreased in animals in the infected groups, regardless of ivermectin treatment, compared with percentage and counts in the control and preinfection groups (Fig. 8c). Moreover, animals in the infected group that received ivermectin treatment showed an increase in the number of neutrophils compared with animals in the infected group (Fig. 8d).

To further characterize the reduction in lymphocytes observed in the animals in the infected groups, B and T lymphocytes were analyzed by the detection of specific cell surface markers, CD19 (B lymphocytes) or CD8 and CD4 (T lymphocytes), at the endpoint of the experiment. The results showed that both B and T lymphocyte percentages were reduced in the mice from the virus-infected groups compared to the control group (Fig. 8b), with CD8 + cells being the subpopulation with the greatest reduction (64% and 66% depletion in the infected and infected + IVM groups, respectively).

### Ivermectin reduces the TNFα levels in infected mice

**Figure 9:**
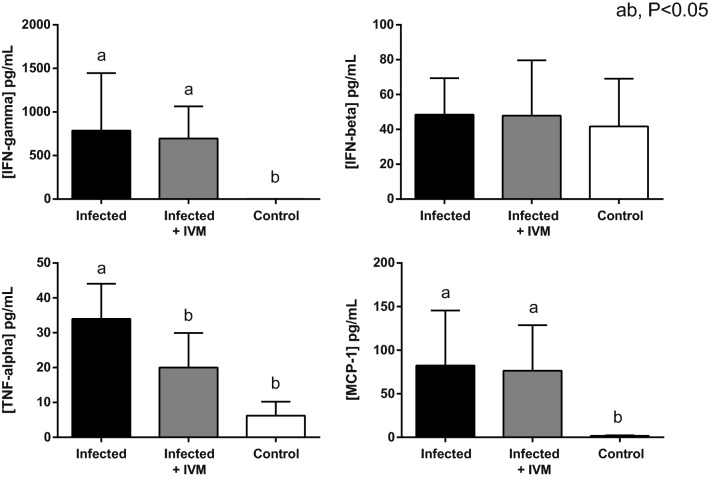
Detection of cytokines in the plasma of all experimental groups (infected, infected + IVM, and control). Murine plasma was obtained 5 days postinfection, and cytokine concentrations were determined by multiplex bead array (mean ± SD). Different letters indicate statistically significant differences (p < 0.05).

Cytokine levels obtained from the plasma samples at the endpoint of the experiment (5 days after viral inoculation) were measured in all three groups. From the panel of 13 inflammatory-related cytokines, only IFNγ and MCP-1 were significantly increased in both infected groups (p < 0.05) compared with the control group, regardless of ivermectin treatment. On the other hand, TNFα values, which were increased in the mice from the infected group, were reduced in the animals that received ivermectin treatment, with the latter showing no significant difference compared to the control mice (Fig. 9).

No statistically significant differences were observed for the other analyzed cytokines (IL-1b, IL-1a, IL-23, IL-12p70, IL-10, or IFNβ), and some of these proteins (IL-6, IL-27, IL-17A and GM-CSF) were below the detection limit of the assay.

## Discussion

This study proposes a mouse experimental model for the in vivo evaluation of pharmacological therapies against coronavirus diseases. It is well known that preclinical animal models are of utmost importance when developing new therapies or vaccines that will be applied to humans. In addition, the need to develop animal models to study SARS-CoV-2 has been recently proposed by many researchers^23^. Our study is based on the previously tested in vitro reports of the use of ivermectin against several RNA and DNA human and animal viruses^12^, such as influenza A, West Nile, Venezuelan equine encephalitis, Zika, chikungunya, Newcastle disease, and porcine reproductive and respiratory syndrome viruses; HIV-1; dengue fever, yellow fever, tick-borne encephalitis, and pseudorabies viruses; porcine circovirus, parvovirus and bovine herpesvirus. However, most of these studies reported only in vitro results, and information on the effect of ivermectin used in vivo is scarce.

In our model, mice infected with MHV and immediately treated with ivermectin showed a lower hepatic viral load five days after infection and better general health than infected animals not treated with ivermectin. A possible explanation for the lower viral load in the infected animals that received ivermectin may be related to impairment of virus replication in the cells, since this drug has been shown to inhibit nuclear import through inhibition of IMP α/β1 heterodimer formation^24^. In the necropsy and histological analysis, the livers of infected and untreated mice showed the worst appearance, with several animals showing severe hepatocellular necrosis and lymphoplasmacytic inflammatory infiltration. The treated group showed a lower severity of anomalies, although the liver and spleen weights were heavier in the mice in both infected groups than in the mice in the control group. The organ weight increase following infection is indicative of the immune reaction^25^, something that cannot be evaluated in an in vitro model. These findings are typical for MHV-infected mice^26^, and their generalized immune reaction are confirmed with the cytokine levels found in our study in both infected groups.

Serum biochemistry of the liver and kidney showed a clear impairment of the metabolic profile, mainly due to liver damage. Both groups of infected mice showed hypoalbuminemia and hyperglobulinemia, with a decrease in the A/G ratio compared with the control group. Variations in both proteins reflect hepatic function^27^, and the serum concentrations of transaminases such as AST and ALT were significantly higher in the virus-infected mice that did not receive ivermectin and were associated with other variables indicative of liver damage^28^. A considerable decrease in serum creatinine levels was found in the infected mice, representing major liver damage and diminished kidney function in the sick animals. Glucose levels were significantly decreased in the infected mice, probably due to the animals’ loss of appetite related to the impairment of their general health status. Interestingly, this metabolic profile showed major liver damage in the infected animals, in accordance with the remainder of the data analyzed during this study. Treatment with ivermectin was effective in reducing the effects of the viral infection, supporting the use of this model to test novel therapies against coronavirus diseases.

The most relevant hematological findings were an increase in neutrophil and monocyte percentages and a reduction in WBC and lymphocyte counts (B and T) in both infected groups, regardless of ivermectin treatment. Neutrophilia and lymphopenia have been well documented in viral respiratory infectious diseases in mouse models and humans^29–31^. The increase in the percentage of neutrophils in the virus-infected groups may be associated with acute-phase viral infection. On the other hand, the reduction in lymphocytes might be due to migration/retention of these cells in the liver and/or lymphoid tissue.

The rapid development of lymphopenia has also been observed in COVID-19 patients with adverse outcomes, whereby CD4 + T-cells were more severely reduced than CD8 + T-cells^32,33^. The neutrophil count was the only hematological parameter that differed among the virus-infected animals, being higher in the mice from the ivermectin-treated group. Nevertheless, this difference did not impact the WBC differential. Moreover, in both infected groups, the neutrophil counts increased compared with those in the corresponding preinfection timepoint, and ivermectin treatment alone did not lead to numbers that differed from those of the control (data not shown).

Taking the hematological data together, it seems that the differences observed between groups are related to the viral infection itself, not to the effect of ivermectin. The results from studies on the immunomodulatory effects of ivermectin vary^34^, making it difficult to clearly characterize its function. In this regard, this in vivo mouse model of MHV infection would not support a modulatory action of ivermectin on the immune response. On the other hand, these results are in accordance with various reports demonstrating that the broad-spectrum antiviral potential of ivermectin against several RNA viruses is due to its ability to specifically bind to and destabilize the importin α/β heterodimer, thereby preventing importin α/β from binding to the viral protein, which in turn blocks the nuclear trafficking of viral proteins^13,35,36^.

The cytokine analysis showed that only IFN-ɣ and MCP-1 levels were increased in the mice from the virus-infected groups compared to those in the control group. These increases are in line with the general immune response associated with a viral infection. On the other hand, ivermectin treatment did not seem to exert a significant effect on the modulation of most inflammatory cytokines. An exception was TNF-α, whose value was significantly reduced in the ivermectin-treated animals compared with the mice in the infected group. It has been reported that ivermectin can exert anti-inflammatory effects in in vitro cell models by downregulating NF-kB signaling pathways and regulating TNF-α, IL-1β and IL-10^37^, and in in vivo models, it exerts an effect by decreasing the production of TNF-α, IL-1β and IL-6^38^.

In the present study, neither the IL-1β, IL-10 nor IL-6 levels were modulated by ivermectin. It is possible that differences regarding the experimental model, the route of infection and the time window of the measurements can account for these discrepancies, since in a living organism, the immune response is influenced by more than one cellular component of the immunological system. Moreover, the similar hematological profiles of both infected groups suggests that the main antiviral effect of ivermectin does not occur through immunomodulatory actions.

## Conclusion

This study demonstrates that a single dose of ivermectin administration reduces the MHV liver viral load in infected mice, enhancing their general health status. This preclinical model is suitable for further study of the effect of ivermectin against coronavirus infections as a possible surrogate model, facilitating the discovery of available treatments for other coronavirus-related diseases.

## Materials and methods

### Ethics statement

The experimental protocols were approved by the institutional *Comisión de Ética en el Uso de Animales* (CEUA) (protocol #008-16) and were performed according to national law #18.611 and relevant international laboratory animal welfare guidelines and regulations. In addition, the ARRIVE guidelines were followed to carry out the present study.

### Animals and management

A total of 56 BALB/cJ female mice (6–8 weeks old) were bred at the Transgenic and Experimental Animal Unit of Institut Pasteur de Montevideo under specific pathogen-free conditions in individually ventilated racks (IVC, 1285L, Tecniplast, Milan, Italy). During the experimental procedure, the mice were housed in groups of seven in negative pressure microisolators (ISOCageN, Tecniplast) with aspen chip wood bedding (Toplit 6, Safe, Augy, France), paper towels and cardboard tubes for environmental enrichment. They had ad libitum access to autoclaved food (5K67, LabDiet, MO, US) and filtered water. The housing environmental conditions during the experiment were as follows: 20 ± 1 °C temperature, 30–70% relative humidity, negative pressure (biocontainment) and a light/dark cycle of 14/10 h. All procedures were performed under Biosafety level II conditions. The mice were randomly distributed into three experimental groups: infected (n = 20), infected + IVM (n = 20) and control (n = 16) groups. Experiments were conducted in three independent replicates.

### MHV-A59 preparation

MHV-A59 (ATCC VR-764) viruses were expanded in murine L929 cells (ATCC CCL-1) to reach a concentration of 1 × 10^7^ plaque forming units (PFUs)/mL. The virus-containing supernatants were stored at -80 °C until further use.

### Infection and treatment

Before infection, the mice were weighed, and blood samples were obtained from the submandibular vein to determine the basal blood parameters. The mice were infected with 6,000 PFU of MHV-A59 diluted in 100 µL of sterile PBS administered by the intraperitoneal route. Immediately thereafter, mice in the infected + IVM group were treated with a single s.c. dose of 500 µg/kg ivermectin (Ivomec 1%, Merial, Paris, France) diluted in 50 µL of PBS. The mice in the other two groups (infected and control) received 50 µL of PBS s.c.

Five days after infection/treatment, the mice were weighed, and 300 µL of blood was retrieved from the submandibular vein for plasma cytokine quantification and metabolic and hematological profiles. The mice were immediately euthanized by cervical dislocation to dissect the liver and spleen for weight recording and histological and qPCR analysis. At necropsy, the liver appearance was blindly scored (0 to 3) by an independent and trained technician who considered the main pathologic pattern of the MHV infection^39,40^. Briefly, gross hepatic lesions were identified as multifocal to coalescent whitish spots of less than 1 mm diameter and were defined as hepatic granulomas.

### Histological analysis

Immediately after necropsy, the liver and spleen were fixed in 10% neutral buffered formalin (pH 7.4) for further processing. For evaluation, they were embedded in paraffin, sectioned in 4 µm sections and stained with hematoxylin–eosin (H&E) according to Kyuwa et al*.*^41^. Whole specimens were examined under a light microscope (BX41, Olympus, Tokyo, Japan) at 10 × in three randomly selected areas or in the highest incidence areas of each specimen by three different pathologists to establish a histopathological score in each case. The score was determined on the basis of a previously defined semiquantitative microscopy grading scale representing typical histopathologic changes caused by MHV (characterized by the presence of hepatocellular necrotic areas and granulomatous inflammatory reaction) according to the following criteria: 0 = normal (no necrotic areas identified in the whole specimen); 1 ≤ 10 necrotic areas; 2 = 10–20 necrotic areas; and 3 ≥ 20 necrotic areas.

### Hepatic viral load

After dissecting and trimming the whole liver, samples (0.5 × 0.5 cm) of the hepatic right lobe were retrieved for qPCR analysis. The samples were loaded in cryotubes with TRI Reagent (Sigma-Aldrich, Saint-Louis, MO, US), immediately plunged into liquid nitrogen and stored at − 80 °C until analysis. Total RNA was isolated according to the manufacturer’s instructions. cDNA was synthesized from 2 µg of total RNA using M-MLV Reverse Transcriptase (Thermo Fisher, Waltham, MA, US) and random primers (Invitrogen, Carlsbad, CA, US). Sample analysis was performed with a QuantStudio 3 Real-time PCR system (Thermo Fisher) using FastStart Universal SYBR Green Master (Rox) (Roche, Basel, CH). The primer sequences were as follows: MHV forward primer (5′–3′): GGAACTTCTCGTTGGGCATTATACT and MHV reverse primer (5′–3′): ACCACAAGATTATCATTTTCACAACATA. The reactions were performed according to the following settings: 95 °C for 10 min and 40 cycles of 95 °C for 15 s and 60 °C for 1 min. The quantification of the viral load was performed with the relative standard curve method using an undiluted positive control of 10^6^ arbitrary units (AU).

### Blood biochemistry profile

Individual whole blood (100 µL) was analyzed for liver and kidney biochemical profiles using a POINTCARE V2 automatic device (Tianjin MNCHIP Technologies Co., China) at the beginning (preinfection determination) and at the end of the experiment (postinfection determination). The analyzed parameters included total proteins (TP), albumin (ALB) level, globulin (GLO) level, ALB/GLO ratio, total bilirubin (TBIL) level, alanine aminotransferase (ALT) level, aspartate aminotransferase (AST) level, gamma glutamyl transpeptidase (GGT) level, blood urea nitrogen (BUN) level, creatinine (CRE) level, BUN/CRE ratio and glucose (GLU) level.

### Hematological parameters

For hematologic analysis, aliquots of 20 µL of blood were collected and stored in 0.5 mL microtubes containing EDTA potassium salts (W anticoagulant, Wiener Lab, Rosario, Argentina) at a ratio of 1:10 (EDTA:blood) during the pre- and postinfection stages. All measurements were performed within four hours after blood collection. Total WBC count, differential WBC count and percentage, RBC count, HGC level, HCT level, and PLT count were evaluated using an autohematology analyzer BC-5000Vet (Mindray Medical International Ltd., Shenzhen, China).

### Cytokine quantification and flow cytometry analysis

Bead-based multiplex assays were employed to quantify cytokines (LEGENDplex mouse Inflammation Panel, BioLegend Inc., San Diego, CA, US) in plasma samples obtained from the mice pre- and postinfection, according to the manufacturer's instructions. Briefly, blood samples were combined with EDTA as an anticoagulant and centrifuged for 10 min at 1000×g, and plasma was recovered and stored at − 20 °C until use. For the assay, 25 µL of twofold diluted plasma samples, diluted standards, and blanks were added to the corresponding tubes; 25 µL of premixed beads and detection antibodies were added to all of the tubes. The tubes were incubated for 2 h at room temperature with shaking. Without washing the samples, 25 µL of streptavidin–phycoerythrin (SA-PE) conjugate was added, and the tubes were incubated for 30 min. Then, the samples were washed and suspended in 200 µL of wash buffer. The data were acquired with a BD Accuri C6 flow cytometer (BD Biosciences, CA, US). BD Accuri C6 software was used for data acquisition. Bead excitation was achieved using 488 and 640 nm lasers, and the emission was detected using 530/30 and 665/20 nm bandpass filters, respectively. For each analyte to be detected, 4,000 beads gated on a forward scatter (FSC) versus side scatter (SSC) were recorded by dot plot. The data were processed with BioLegend LEGENDplex Data Analysis Software. The results represent the concentration expressed in pg/mL.

### B and T lymphocyte analysis by flow cytometry

Lymphocyte surface markers were evaluated in peripheral blood samples (50 μL) anticoagulated with EDTA. Erythrocytes were removed by suspending cells in 1 mL of lysis buffer (155 mM NH_4_Cl, 12 mM NaHCO_3_, 0.1 mM EDTA, pH 7.4) for 10 min at room temperature. After washing in PBS containing 0.2% bovine serum albumin, nucleated cells were incubated on ice for 15 min with an antibody mixture. The following fluorophore-conjugated antibodies were used: anti-CD4-FITC (#11,004,181, clone GK1.5) and anti-CD8-PE-Cy7 (#25,008,182, clone 53–6.7) from eBioscience (San Diego, CA, US) and anti-CD19-PerCP-Cy 5.5 (#551,001, clone ID3) from BD Pharmingen (San Diego, CA, US). Flow cytometry analysis was performed using an Attune Nxt Acoustic Focusing Cytometer (Thermo Fisher) equipped with a 488 nm laser. Emissions were detected using 530/30, 695/40 and 780/60 nm bandpass filters for FITC, PerCP-Cy5.5 and PE-Cy7, respectively. FlowJo software, version 10.6.1 (Tree Star, Ashland, Oregon, US), was used for data analysis. Unstained controls, single-color controls and fluorescence-minus-one controls were used to establish baseline gate settings for each respective antibody combination.

Lymphocytes were gated based on their FSC and SSC dot plot profiles, and an FSC area vs FSC height dot plot was used to exclude doublets. B lymphocytes were defined as CD19-PerCP-Cy5.5-positive cells. For T lymphocyte analysis, a gate was placed on the CD19-negative population, and based on the PE-Cy7 vs FITC dot plot, CD8-PE-Cy7-positive cells and CD4-FITC-positive cells were defined as CD8 + and CD4 + lymphocytes, respectively. A minimum of 10,000 events in a single cell region were collected. The results are expressed as percentage of the specific cell type from the analyzed single-cell population.

### Statistical analysis

Statistical analysis was performed using generalized linear mixed models (GLMM, InfoStat software^42^), which included the treatments (three groups) and time (pre- and postinfection) as fixed variables and the animals and replicates as random variables. The data for continuous variables were evaluated for normality and homogeneity of variance by histograms, q-q plots, and formal statistical tests as part of the univariate procedure. The type of variance–covariance structure was chosen depending on the magnitude of the Akaike information criterion (AIC) for models analyzed by heterogeneous compound symmetry, unstructured, autoregressive, spatial power, and first-order interdependence methods. The model with the lowest AIC was chosen. The data are presented as the means ± SEM, and the significance level was defined as a *p*-value of 0.05.

## Data Availability

All data generated or analyzed during this study are included in this published article.
